# EMFSA: Emoji-based multifeature fusion sentiment analysis

**DOI:** 10.1371/journal.pone.0310715

**Published:** 2024-09-19

**Authors:** Hongmei Tang, Wenzhong Tang, Dixiongxiao Zhu, Shuai Wang, Yanyang Wang, Lihong Wang

**Affiliations:** 1 School of Computer Science and Engineering, Beihang University, Beijing, China; 2 Xinjiang Astronomical Observatory, Chinese Academy of Sciences, Urumqi, China; 3 School of Aeronautic Science and Engineering, Beihang University, Beijing, China; 4 Jiangxi Research Institute of Beihang University, Nanchan, China; 5 National Computer Network Emergency Response Technical Team/Coordination Center of China, Beijing, China; Centro de Investigacion en Ciencias de Informacion Geoespacial AC (Research Center on Geospatial Information Sciences), MEXICO

## Abstract

Short texts on social platforms often suffer from insufficient emotional semantic expressions, sparse features, and polysemy. To enhance the accuracy achieved by sentiment analysis for short texts, this paper proposes an emoji-based multifeature fusion sentiment analysis model (EMFSA). The model mines the sentiments of emojis, topics, and text features. Initially, a pretraining method for feature extraction is employed to enhance the semantic expressions of emotions in text by extracting contextual semantic information from emojis. Following this, a sentiment- and emoji-masked language model is designed to prioritize the masking of emojis and words with implicit sentiments, focusing on learning the emotional semantics contained in text. Additionally, we proposed a multifeature fusion method based on a cross-attention mechanism by determining the importance of each word in a text from a topic perspective. Next, this method is integrated with the original semantic information of emojis and the enhanced text features, attaining improved sentiment representation accuracy for short texts. Comparative experiments conducted with the state-of-the-art baseline methods on three public datasets demonstrate that the proposed model achieves accuracy improvements of 2.3%, 10.9%, and 2.7%, respectively, validating its effectiveness.

## Introduction

An emotion refers to an attitude, thought, or judgment caused by sensations [1]. Emotions are generally considered to have three polarities: positive, negative, and neutral. Sentiment analysis involves computationally processing the ideas, emotions, and subjectivity in a text [2]. Currently, social media platforms host vast, diverse, and mixed-modal data. An analysis of platforms such as Twitter reveals an abundance of short text messages, hashtags, and emojis. Nearly half of the content on Instagram includes emojis [3], and Facebook sees the use of 5 billion emojis every day. Due to the presence of unconstrained language, sparse features, evident fragmentation, limited vocabularies, high noise levels, colloquial expressions, and implicit opinions and attitudes in syntactic structures and contextual clues within short text messages on social platforms, emotional expressions become ambiguous. Although text-based opinion mining methods have proven highly useful in sentiment analysis tasks, they still face issues such as domain, topic, and time dependencies [4].

In 1997, emojis appeared in Japan, providing visual representations of emotions through nonverbal cues. They serve as intentional carriers of emotional states and are widely used in online communications. And in the context of media communications, Daft et al. [5] suggested that higher information processing efficiency is achieved when multiple cues are available. Approximately 20 billion tweets are posted daily on platforms such as Twitter, and with each new Unicode version, new emojis are introduced, making them increasingly relevant to sentiment analysis tasks [6]. Emojis can provide contextual information, enhancing the ability to process oral information content. To address issues such as insufficient semantic expressions, sparse semantic features, and polysemy in short text sentiments, this paper proposes an emoji-based multifeature fusion sentiment analysis (EMFSA) model. The model primarily explores the combination of various features, including emojis, topics, and text, for mining sentiments across different modalities. It utilizes a cross-attention mechanism to integrate these features, obtaining complementary and enhanced information between modalities. This approach facilitates matching similar contexts and better recognizing and understanding the meanings of contextual representations and attention, ultimately improving the accuracy of sentiment analysis. Our contributions are as follows:

(1) An emoji-enhanced text feature extraction and pretraining method is proposed. The exBERT model is introduced as the base framework, an encoder (emo_exBERT) is designed for synchronously extracting text and emoji features, and the model is pretrained on a large corpus containing emojis. This approach aims to extract emoji-based contextual semantic information, enhancing the overall semantic representation of the given text.
(2) A sentiment- and emoji-masked language model (Senti_MLM) that is suitable for English text is introduced. This model prioritizes masking emojis and words with implicit sentiments, yielding a higher masking probability. The objective is to focus on learning semantic representations, thereby enhancing the effectiveness of the subsequent sentiment analysis process.
(3) A multifeature fusion method based on a cross-attention mechanism is proposed. A biterm topic model is introduced to further discover the latent semantic relationships (i.e., topics) implied between documents and words. This process helps determine the importance of each word in the input text from a topic perspective. Subsequently, these importance scores are fused with the original semantic information of emojis and the text features enhanced by emojis, aiming to improve the accuracy of the semantic representations of short-text sentiments and enhance these sentiments with the topics and emojis in the text.
(4) The experimental results show that the model exhibits outstanding performance in sentiment classification tasks conducted on public datasets and outperforms the existing methods.

The remaining sections of this paper are organized as follows: Section 2 examines and discusses the related literature. Section 3 provides a detailed introduction to the EMFSA model proposed in this paper. Section 4 discusses the experimental setups, comparison methods, and implementation details and presents quantitative and qualitative evaluations of the experimental results. Finally, in Section 5, we summarize the findings of this study and outline our future work.

## Related work

### Sentiment analysis

Sentiment analysis is performed mainly using lexicon-based methods [7–10] and machine learning-based methods [11–13] to analyze document-level sentiments. However, the obtained information is often incomplete, leading to suboptimal accuracy. With the development of deep learning techniques, Wang et al. [14] proposed an attention-based LSTM combination to achieve improved model performance. However, LSTM is time-consuming to train and can encode only unidirectional sequence information. Subsequently, researchers were inspired by the excellent performance of graph neural networks (GNNs) in terms of managing the complex relational structures in text and preserving the global information contained in feature embeddings, which led to better classification results [15]. However, neural network-based approaches still suffer from insufficient model training processes and poor generalization performance. Thereafter, researchers attempted to solve the sentiment analysis problem using two approaches based on BERT models. First, this approach mainly employs the pretrained model + fine-tuning strategy to directly fine-tune the pretrained BERT model according to the sentiment analysis task, and it has produced impressive results in various natural language processing tasks [16–20]. Second, the masked language modeling (MLM) approach is used to further pretrain on domain-specific datasets and then fine-tune on them for sentiment analysis tasks [21, 22]. This approach involves retraining BERT again and then fine-tuning it for fine-grained sentiment analysis and other sentiment-related tasks. However, no special attention is given to “sentiments” throughout the pretraining process of the above “retraining” approach. Subsequently, multiscale graph attention networks (MSGATs) [23, 24] based on dependency grammars and hybrid models based on topic knowledge [25] were proposed. However, due to the sparse semantic representations and inadequate semantic expressions of short texts, these models face challenges when addressing emerging terms on social media networks and realizing high-precision sentiment analysis.

### Sentiment analysis with emojis

Emoji-based sentiment analysis methods are divided into three main categories: dictionary-based methods, machine learning-based methods, and deep learning-based methods. Dictionary-based methods focus on building emoji sentiment dictionaries to support text sentiment analysis tasks. Kralj et al. created the first emoji sentiment dictionary [26]. Subsequently, the Emoji2Vec [27] pretrained embedding method was developed, and EmojiNet (a semantic repository of emojis) was published [28]. Researchers created raw sentiment lexicons [29] and emoji lexicons using Unicode Consortium classification [30], unsupervised classification [6], manual compilation [31], and synonym categorization [32, 33] to automate the creation of combined lexicons [34]. This method lays the foundation for research on emoji sentiment analysis. Although this method is simple and effective, its rules may not fully cover all possible situations. Machine learning methods involve training sentiment classifiers based on corpora to analyze the sentiments of text [35]. However, this method is sensitive to the input data representation, requires the computation of prior probabilities, and achieves low accuracy in sentiment analysis tasks.

Deep learning methods excel at effectively utilizing contextual word embeddings to generate dense document representations, thereby significantly improving model accuracy. Examples include attention-based network models [36–38], BiLSTM [39], deep neural networks, models that combine emoji- and lexicon-based sentiment enhancement with fuzzy inference [40], document representation models that incorporate word-sentiment associations and topic models [41], symbiotic graph networks [42] (which learn emoji representations by embedding them into emoji nodes based on the semantic information derived from an external knowledge base, such as EmojiNet to learn emoji representations with an up-to-date emoji-text design baseline), and recurrent neural network models [43, 44]. However, these approaches explore sentiments only from the textual and emoji perspectives and do not consider the influence of topics on sentiment analysis. To further explore the potential semantic relationships between documents and words, topic modeling-based approaches have been used in sentiment analysis studies [45, 46]. Haque et al. [47] used latent Dirichlet allocation (LDA)-based topic modeling to identify trending topics in ChatGPT-related tweets and performed a manual labeled sentiment analysis. Taecharungroj [48] explored the capabilities and weaknesses of ChatGPT using LDA-based topic modeling. These approaches validate the effectiveness of topic embedding in sentiment analysis tasks but lack the ability to analyze short textual scenarios with sparse semantics. This study focuses on the short text included in mixed-mode data containing emoticons on social media platforms. To address the problem of sparse word co-occurrence patterns in individual documents, this paper adopts the BTM short text topic model [49] based on word co-occurrence patterns to capture the entire text passage and the topic vectors of each token in it; the shared topic distribution is then trained across the corpus.

With the continuous development of pretrained large-scale language models, researchers have applied these models to sentiment analysis tasks. In 2017, the transformer structures [50] of deep learning models surpassed 100 million parameters. The parameters of the BERT network model [51] exceeded 300 million for the first time, and the parameter counts of models such as LLaMA [52], the GLM [53, 54], and GPT-3 [55] exceeded tens of billions. Large-scale language models are perfect combinations of big data, high computing power, and advanced algorithms. Studies have shown that they greatly enhance the pretraining and language generation capabilities of large models in general domains. Pradhan et al. [56] proposed semantic attention optimization, stacking, bidirectional gated recurrent unit with semantic attention (SRBi-GRU-SA), and multichannel word embedding models to represent a text by combining sentiment information with semantic information obtained from a natural language model (BERT). Nusrat et al. [57] proposed a converter-based approach for emoji prediction using BERT. Talaat et al. [58] proposed four deep learning models that combined BERT with bidirectional long short-term memory (BiLSTM) and bidirectional gated recurrent unit (BiGRU) algorithms. Yang et al. [59] proposed combining pretrained BERT models with temporal convolutional networks (TCNs) and graphical convolutional networks for short text classification and emoji prediction. In addition, generative dialog models such as ChatGPT, which can capture complex linguistic patterns, have been investigated in sentiment analysis studies. Mithun et al. [60] investigated the weaknesses of the ChatGPT model in terms of detecting emoji-based hate speech. In cases involving emoji-based hate speech, the model performs poorly when positive emojis are used in hate posts and fails to accurately label non-English-language inputs. However, the aforementioned methods only utilize features acquired from text and emojis without considering the introduction of topic features for sentiment analysis purposes. Additionally, how to handle the continuous emergence of new network terms on social media platforms has not been considered.

According to the analysis of a literature review, the current emoji-based sentiment analysis method mainly adopts pretrained models. A pretrained model based on the transformer architecture utilizes a self-attention mechanism to achieve fast parallelism and increases the network depth to obtain additional global information. For scenarios in which new vocabulary emerges, to further solve the problems concerning sparse semantic features and insufficient sentiment expressions in short texts while considering the training cost, model efficiency, and model performance factors, this study proposes adopting a new neural network model called exBERT [61] (represented by transformers) as the foundation. This study focuses on extracting and combining features derived from text, topics, and emoticons and improving the sentiment accuracy rate achieved by conducting fine-tuning in specific domains.

## Methodology

### Preliminaries

Given a short textual passage that includes some emojis, such as “Nick has such cute balls 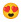

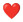
”, sentiment analysis is required to determine whether the sentiment orientation is positive, negative or neutral. Formally speaking, this task can be expressed as a function F:T↦P, which takes a short textual passage *T* as its input and returns *P*, indicating the probability that the sentiment tendency of *T* is positive, negative or neutral.

In mainstream approaches, a pipeline consisting of an encoder E and a classifier C is employed to solve sentiment analysis task, i.e., F=E°C. The encoder is used to transform a textual passage *T* into an embedding *E*, and the classifier outputs a probability *P* according to *E*. Generally, the embedding *E* can be a *d*-dimensional vector that represents all the semantics of *T* or a sequence consisting of *N* *d*-dimensional vectors that represent each of the *N* words in *T*.

The encoder is more significant because of its role in semantic comprehension, and in our work, the encoder is enhanced with the emoji semantics and topics so that it can yield text embeddings of higher quality to improve sentiment analysis.

### EMFSA model

**Fig 1:**
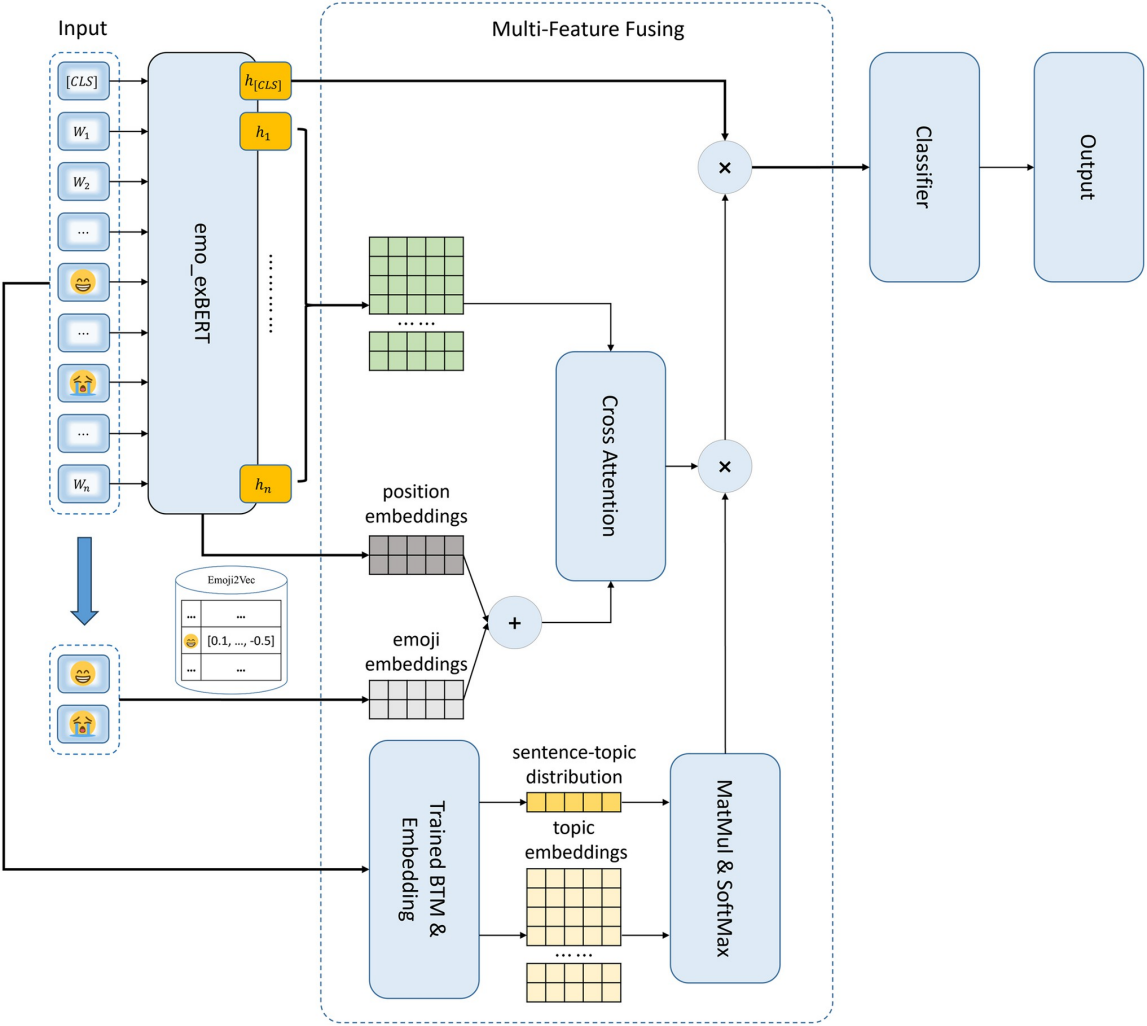
The overall architecture of our EMFSA model.

The overall architecture of our EMFSA model is shown in Fig 1. The process starts with the joint feature extraction module (emo_exBERT), which can capture the long-distance contextual dependencies between text and emojis to obtain all the semantic features of the input with emojis for the subsequent sentiment analysis task. In the domain-specific additional pretraining stage of emo_exBERT, we propose a novel sentiment word priority-based masked language model (Senti_MLM), which masks emojis and words with richer sentiments, exhibiting a higher probability of prioritizing the learning of semantic representations and improving the effect of the downstream sentiment analysis process.

Because textual passages on social platforms are usually short and casual in terms of their expressions, they contain insufficient semantic information and excessive noise; to address these issues, we inject two auxiliary pieces of information: descriptions of emojis and descriptions of topics. For the former, we use Emoji2Vec [27], which trains emoji representations on the basis of text descriptions to embed the emojis; these embeddings are expected to enhance the semantics of textual passages with emojis. For the latter, a biterm topic model (BTM) [49] is trained and subsequently used to extract the topic feature of the whole input sentence and each word in it. By comparing the topic consistency levels of words and their corresponding sentences, we can learn which words are more significant, and greater emphasis is placed on the semantics of these words so that the impact of noise can be mitigated. Finally, we design a multifeature fusion module employing a cross-attention mechanism to fuse the features extracted by emo_exBERT and the two auxiliary information sources; the output of this module is fed into a classifier composed of a fully connected layer, yielding the ultimate sentiment analysis result.

### Joint feature extraction module (emo_exBERT)

**Fig 2:**
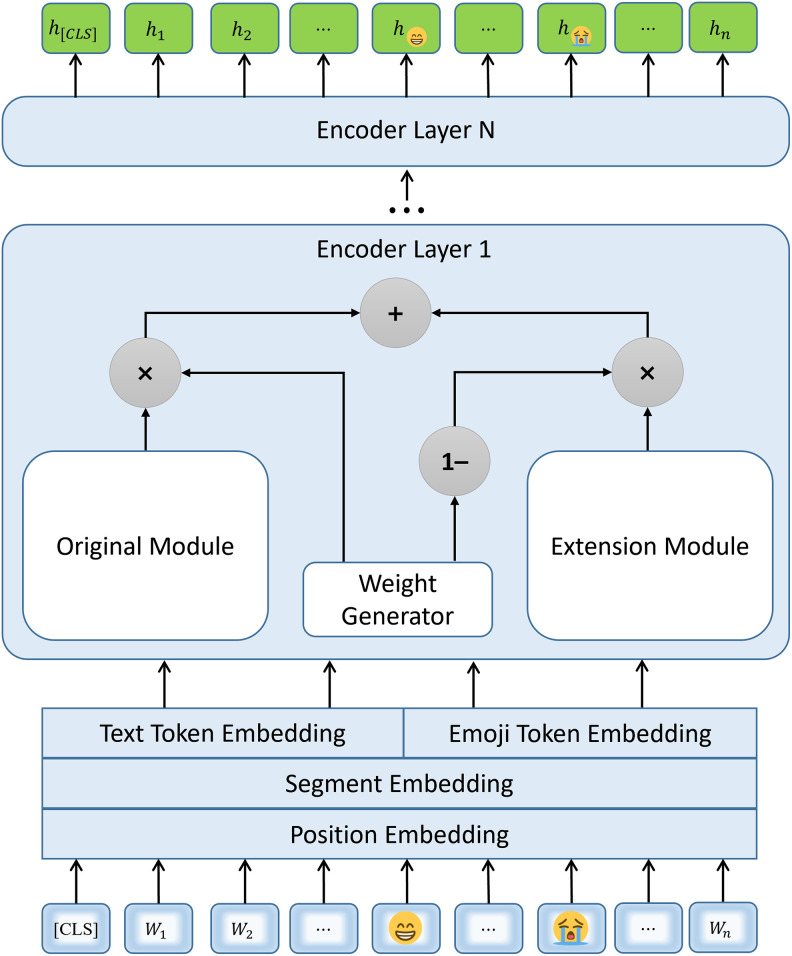
Structure of the joint feature extraction module (emo_exBERT).

Abundant research has indicated that high-quality semantic representations of text can be obtained through pretrained language models (PLMs), thereby enhancing the performance achieved in various downstream tasks. However, the majority of the current PLMs are incapable of recognizing emojis, which poses a challenge when semantically representing text containing emojis. Inspired by exBERT [61], which extends the pretrained BERT model with domain-specific vocabulary and has been validated in the biomedical domain, we propose a joint feature extraction module, emo_exBERT, which represents the semantics of text and emojis simultaneously. As shown in Fig 2, the module has the same structure as that of exBERT, which employs a pretrained BERT model as its backbone and introduces two modifications. (1) An extra token embedding layer is added to embed domain-specific tokens (i.e., emojis here). (2) An expanded multihead self-attention submodule is added to each encoder layer, the output of which is the weighted sum of the outputs of the original submodule and the extended submodule. During the adaptive pretraining phase, only the parameters of the emoji token embedding layer, the extension submodule and the weight generator need to be updated, while the remaining parameters are frozen.

Different from exBERT, we use a corpus that includes emojis to pretrain our emo_exBERT module for learning the features of emojis and the interactions between text and emojis. Given a text passage *T* = {[*CLS*], *w*_1_, *w*_2_, ⋯, *w*_*n*_}, where *w*_*i*_ can be a normal word or an emoji, and [*CLS*] indicates the beginning of *T*, emo_exBERT module transforms the passage into a feature sequence *S* = {*h*_[*CLS*]_, *h*_1_, *h*_2_, ⋯, *h*_*n*_}.

### Sentiment word priority-masked language model (Senti_MLM)

Masked language models (MLMs) are often used in the pretraining stages of pretrained language models such as BERT. Such a model randomly masks some tokens contained in the input at a certain proportion and attempts to reconstruct them. Randomness implies equal treatment for each word; however, in sentiment analysis tasks, the contributions of different words usually vary. Therefore, we propose a sentiment word priority masked language model (Senti_MLM), which masks emojis and words possessing richer sentiments with higher probabilities to prioritize learning their semantic representations and thus enhance the performance achieved in the subsequent sentiment analysis task. The specific steps are as follows:

(1) The SentiWordNet sentiment dictionary [7] is queried, and the overall sentiment weight of each token is calculated in the vocabulary of our emo_exBERT module. The overall sentiment weight *sw*_*i*_ of the *i*-th token *w*_*i*_ is shown in Eq (1):

$$\begin{array}{c} sw_{i}=\frac{1}{L}\sum_{l=1}^{L}pw_{i,l}+nw_{i,l}<1 \end{array}$$ (1)

where *L* is the number of meanings for *w*_*i*_ in the SentiWordNet sentiment dictionary, and *pw*_*i*,*l*_ and *nw*_*i*,*l*_ are the positive and negative sentiment weights corresponding to the *l*-th meaning of *w*_*i*_, respectively. If *w*_*i*_ does not exist in SentiWordNet and is not an emoji, *sw*_*i*_ = 0; if it is an emoji, *sw*_*i*_ = 1.

(2) For each batch in the training corpus, its unnormalized masked probability matrix (*mp*_*i*,*j*_) is computed, where *mp*_*i*,*j*_ is the element in row *i* and column *j* of the matrix, which indicates the unnormalized masked probability of the *j*-th token of the *i*-th textual passage in this batch, as shown in Eq (2):

$$\begin{array}{c} mp_{i,j}=\{\begin{array}{cc} 1, & sw_{i,j}=0\\ (1+sw_{i,j})\times smr, & sw_{i,j}>0 \end{array} \end{array}$$ (2)

where *sw*_*i*,*j*_ denotes the overall sentiment weight of the *j*-th token of the *i*-th textual passage in the batch, and *smr* indicates the sentiment word masked coefficient, which is used to increase the relative masked probabilities of sentiment words.

(3) The unnormalized masked probability matrix is normalized so that the expected number of masked tokens in each batch is proportional to the total number of tokens in that batch, with the ratio equal to the predefined normalization ratio (set to 0.15), as shown in Eq (3):

$$\begin{array}{c} {\left(mp_{i,j}\right)}_{n}=Normalize((mp_{i,j}))=(\frac{\#\left(mp_{i,j}\right)\cdot \gamma \cdot mp_{i,j}}{\sum_{i}\sum_{j}mp_{i,j}}) \end{array}$$ (3)

where (*mp*_*i*,*j*_)_*n*_ is the normalized masked probability matrix, *Normalize*(⋅) denotes the normalize function, #(*mp*_*i*,*j*_) indicates the total number of elements in (*mp*_*i*,*j*_), and *γ* is the predefined normalization ratio mentioned above.

**Fig 3:**
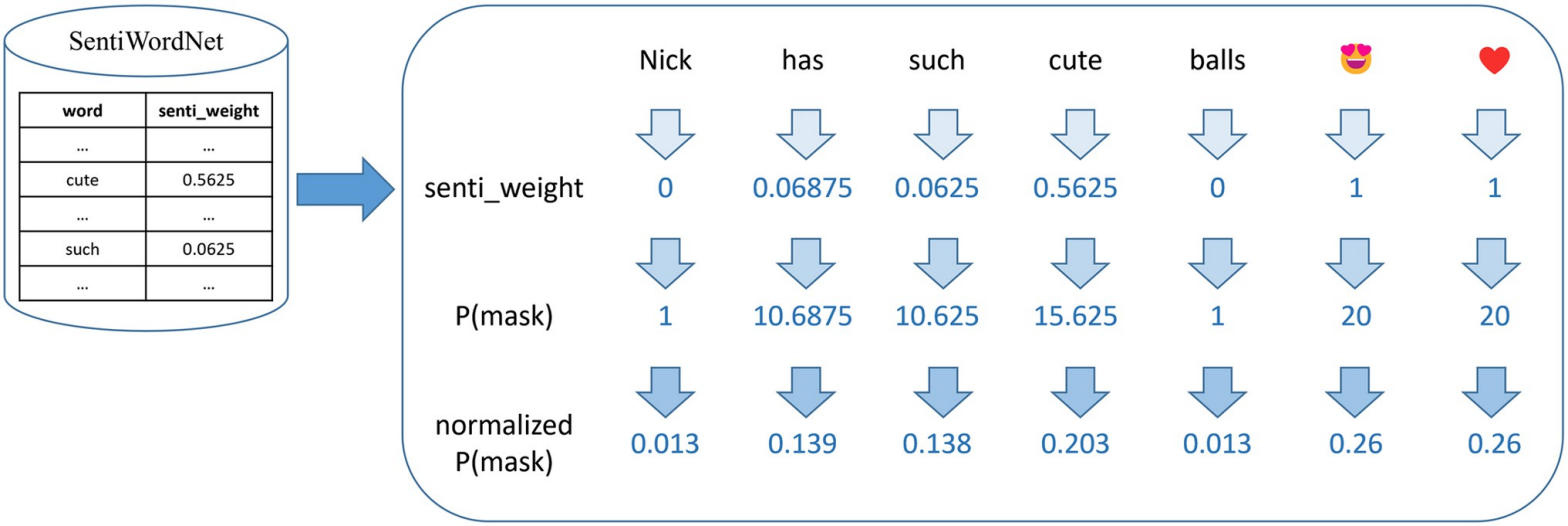
An illustrative example of the sentiment word priority-masked language model (Senti_MLM).

An illustrative example is shown in Fig 3. For a batch that includes only one textual passage, “Nick has such cute balls 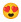

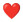
”, we first calculate the overall sentiment weight of each token (assuming that each word is a token; whitespace characters are not illustrated here), i.e., the “senti_weight” row in the right block. Then, we compute the unnormalized masked probability matrix, the result of which is (1 10.6875 10.625 15.625 1 20 20), as shown in “P(mask)” row, and finally normalize the matrix to (0.013 0.139 0.138 0.203 0.013 0.26 0.26), i.e., the “normalized P(mask)” row.

### Multifeature fusion module

This module is aimed at fuse the joint feature of text and emoji extracted by emo_exBERT and two additional features yielded by the introduced auxiliary information, named emoji description semantic feature and topic feature, respectively. Given a textual passage with emojis *T* = {[*CLS*], *w*_1_, *w*_2_, ⋯, *w*_*n*_}, the above two additional features can be obtained as follows:

**Emoji description semantic feature.** We first extract all the emojis in *T* and arrange them in the original order to form a sequence $S_{e}=\left\{w_{\Phi_{1}},\ldots ,w_{\Phi_{m}}\right\}$, where $w_{\Phi_{i}}$ is an emoji, *m* is the number of emojis in *T* and 1 ≤ Φ_1_ < ⋯ < Φ_*m*_ ≤ *n*, and then lookup trained emoji embeddings provided by Emoji2Vec [27] to transform *S*_*e*_ into an emoji embedding matrix $E_{e}={\left(e_{\Phi_{1}},\ldots ,e_{\Phi_{m}}\right)}^{T}$. Considering the impact of the order among the emojis contained in *T* on semantic, we enhance the emoji embeddings using the parameters in the position embedding layer of emo_exBERT, as shwon in Eq (4):

$$\begin{array}{c} E_{e}^{\prime }=E_{e}W_{prj}+E_{p} \end{array}$$ (4)

where $E_{e}^{\prime }$ is the enhanced emoji embedding matrix, i.e., emoji description semantic feature, *W*_*prj*_ is a trainable parameter, and $E_{p}={\left(pe_{\Phi_{1}},\ldots ,pe_{\Phi_{m}}\right)}^{T}$ is the corresponding position embedding matrix.

**Topic feature.** We use biterm topic model (BTM) [49] to capture the topic vectors of the whole input textual passage and each token in it. BTM is a short text topic model based on word co-occurrence patterns. In contrast to LDA [62], which trains a separate topic distribution for each document in the given corpus, BTM trains a shared topic distribution for the entire corpus, thereby resolving the problem concerning the sparse word co-occurrence patterns in individual documents. BTM assumes that the entire corpus is generated through the following process:

(1) For each topic *t*_*k*_ in the topic set (*k* = 1, ⋯, *K*, where *K* is the number of topics), generate a prior distribution $\Phi_{t_{k}}∼Dir\left(\beta \right)$ for its corresponding word distribution.
(2) Generate a prior distribution *θ* ∼ *Dir*(*α*) for the topic distribution of the entire corpus.
(3) For each word co-occurrence pair *b* contained in the corpus, first assign a topic *t* to it and then generate the two words it contains *bw*_1_ and *bw*_2_, where *bw*_1_, *bw*_2_ ∼ *Multi*(Φ_*t*_).

Based on the generation process described above, the likelihood is as follows:

$$\begin{array}{c} P\left(B\right)=\Pi_{(bw_{1},bw_{2})\in B}\sum_{t}P\left(t\right)P\left(bw_{1}|t\right)P\left(bw_{2}|t\right) \end{array}$$ (5)

where *B* is the set of word co-occurrence pairs. By using the Gibbs sampling method to maximize the likelihood, the latent variables *P*(*t*) and *P*(*w*|*t*) can be obtained by Eqs (6) and (7), respectively:

$$\begin{array}{c} P\left(t\right)=\frac{n_{t}+\alpha }{|B|+K\alpha } \end{array}$$ (6)

$$\begin{array}{c} P\left(w|t\right)=\frac{n_{w|t}+\beta }{\sum_{w}n_{w|t}+M\beta } \end{array}$$ (7)

where *n*_*t*_ denotes the number of word co-occurrence pairs assigned to topic *t*, *n*_*w*|*t*_ denotes the number of times word *w* is assigned to topic *t*, *M* is the size of the vocabulary, and *α* and *β* are hyperparameters of the model.

After training the BTM, we calculate the topic vector of the whole textual passage *T*, i.e., the sentence-topic distribution denoted by *TD*, using Eq (8):

$$\begin{array}{c} TD=(P\left(t_{1}|T\right),\cdots ,P\left(t_{K}|T\right)) \end{array}$$ (8)

where

$$\begin{array}{c} P\left(t_{k}|T\right)=normalize_{T}(\sum_{b\in B_{T}}P(t_{k}|b)) \end{array}$$ (9)

$$\begin{array}{c} P\left(t_{k}|b\right)=normalize_{b}\left(P\left(t_{k}\right)P\left(bw_{1}|t_{k}\right)P\left(bw_{2}|t_{k}\right)\right) \end{array}$$ (10)

Here, *bw*_1_, *bw*_2_ ∈ *b*, *B*_*T*_ indicates the set of word co-occurrence pairs in *T*, and *normalize*_*T*_(⋅) denotes the normalization operation such that $\sum_{k=1}^{K}P(t_{k}|T)=1$; *normalize*_*b*_(⋅) is similar to that.

The topic vectors of each token in *T*, i.e., the topic embedding matrix denoted by *TE*, can be obtained by Eq (11):

$$\begin{array}{c} TE=\left(p_{i,k}\right)=\left(P\left(t_{k}|w_{i}\right)\right) \end{array}$$ (11)

where

$$\begin{array}{c} P\left(t_{k}|w_{i}\right)=normalize_{w_{i}}\left(P\left(t_{k}\right)P\left(w_{i}|t_{k}\right)\right) \end{array}$$ (12)

Here, $normalize_{w_{i}}\left(\cdot \right)$ is similar to *normalize*_*T*_(⋅).

The ultimate topic feature *TC* for denoising is the topic consistency between *w*_*i*_ (each token in *T*) and *T*, as shown in Eq (13):

$$\begin{array}{c} TC=softmax(TD\cdot TE^{T}) \end{array}$$ (13)

**Feature fusion.** We fuse the three features of *T*: the joint feature of text and emojis extracted by emo_exBERT *S* = {*h*_[*CLS*]_, *h*_1_, *h*_2_, ⋯, *h*_*n*_}, which can be split into $h_{\left[CLS\right]}^{T}$ and a matrix *S*_¬[*CLS*]_ = (*h*_1_, *h*_2_, ⋯, *h*_*n*_)^T^; the emoji description semantic feature $E_{e}^{\prime }$; and the topic feature *TC*. The fusion operation is completed by Eq (14):

$$\begin{array}{c} F=h_{\left[CLS\right]}^{T}+TC\cdot Attention(S_{\neg [CLS]}W^{Q},E_{e}^{\prime }W^{K},E_{e}^{\prime }W^{V}) \end{array}$$ (14)

where *W*^*Q*^, *W*^*K*^ and *W*^*V*^ are the three weight matrices used in the cross-attention calculation *Attention*(⋅, ⋅, ⋅), which is shown in Eq (15):

$$\begin{array}{c} Attention\left(Q,K,V\right)=sotfmax(\frac{QK^{T}}{\sqrt{d_{k}}})V \end{array}$$ (15)

where *d*_*k*_ is the number of columns of *Q* and *K*.

### Sentiment classification

The fusion feature *F* of the input textual passage *T* is fed into the classifier with a simple fully connected layer, which output the relative sentiment distribution *P* = (*p*_*negtive*_, *p*_*neutral*_, *p*_*positive*_), where *p*_*negtive*_, *p*_*neutral*_ and *p*_*positive*_ indicate the relative probability that the sentiment tendency of *T* is negative, neutral and positive, respectively. Finally, the corresponding sentiment orientation of the maximum among these three probabilities is considered as the ultimate classification result.

We use the cross-entropy loss to optimize the parameters of our EMFSA model, as shown in Eq (16):

$$\begin{array}{c} L=\frac{1}{\left|D\right|}\sum_{T\in D}\;\text{log}\frac{e^{p_{c_{T}}}}{\sum_{c\in C}e^{p_{c}}} \end{array}$$ (16)

where *D* denotes the train set, *C* denotes the label set, which includes three labels: negative, neutral and positive, *c*_*T*_ is the label of *T* and *p*_*c*_ indicates the relative probability that the sentiment tendency of *T* is *c*, output by the above classifier.

## Experiments

The experiments are divided into pretraining and fine-tuning stages. Sentiment- and topic-based pretraining is performed on a larger unlabeled sentiment corpus containing emoticons, and fine-tuning is performed on three public datasets. The following three aspects are described in terms of the utilized datasets and parameter settings: an experimental evaluation, a model performance comparison, and ablation experiments.

### Datasets and setup

Our dataset is consistent with those of Yuan [42] and Nusrat et al. [57, 63]. EmojifyData-EN [64] is a large-scale untagged Twitter dataset, and we restructure it to ensure that at least one emoji is contained in each text of this dataset. SentiWordNet [7] is a lexicon for opinion mining that includes both positive and negative sentiment weights for each lexical sense of each English word. In the experiments, the EmojifyData-EN dataset is used for model pretraining. The Multidomain Sentiment Dataset (MSD) [65], Twitter Dataset (TD) and Emotion Recognition Dataset (ERD) [66] are the main sentiment analysis datasets used to validate the model fine-tuning process. Table 1 provides detailed information about these three datasets.

**Table 1:** Emoji sentiment analysis fine-tuning dataset information.

Dataset	Number of text	Number of text with emoji	Positive	Neutral	Negative
MSD	60K	2212	1405	180	627
TD	162K	1786	816	679	291
ERD	5K	78	34	38	6

In the investigations, only emoji data are used for each dataset. It is ensured that each of the three datasets contains three classifications (negative, neutral and positive), and MSD_Emoji, TD_Emoji and ERD_Emoji are constructed accordingly. The data of this study are preprocessed using the same procedure as that applied by Padmaja et al. [67]. Comprehensive validation of the model’s effectiveness is conducted through five-fold cross-validation on these three public datasets, ensuring that each fold maintains a consistent data distribution, with benign and malignant samples evenly distributed. The architecture is implemented in PyTorch library on a workstation with an Intel Core i-7 and an NVIDIA GeForce RTX 3090 GPU. The accuracy, macro-precision, macro-recall, and macro-F1 metrics are used to evaluate the performance of the model.

### Model performance comparison

To evaluate the performance of the proposed model, we select representative baseline methodologies. TextCNN [68] employs two CNNs to address multilabel classification problems. This approach significantly outperforms the conventional support vector classification (SVC)-based method. The AttBiLSTM [36] model uses a neural attention mechanism with a bidirectional long short-term memory network (BiLSTM) to capture the most essential semantic information in a sentence. Based on the Emojis-Attention and BiLSTM models, EA-BiLSTM [37] was the first method to utilize an attention model to capture the effect of emoticons on the affective polarity of text. The epistemic symbol-based coattention network (ECN) [69] is an emoji-based coattention network used to learn the mutual sentiment semantics between text and emojis on microblogs; this method outperforms numerous baselines in sentiment analyses of brief social media texts. EmoGraph2vec [42] learns emoji representations by constructing cooccurring graphical networks from social data and expanding the external EmojiNet knowledgebase of enriched semantic information to embed emoji nodes. The model creates cutting-edge networks for emoji-containing texts. Our model is compared to the aforementioned baseline methods in terms of accuracy, and an experimental validation conducted on publicly available datasets demonstrates that our method is superior for performing sentiment analysis tasks on text data containing emojis. Table 2 details the classification accuracies attained by the baseline models on the test datasets.

**Table 2:** Classification accuracy of the baseline model on the datasets.

model	Classification accuracy		
	MSD_Emoji	TD_Emoji	ERD_Emoji
TextCNN	0.8258	0.7202	0.6719
Att-BiLSTM	0.8243	0.7198	0.6700
EA-Bi-LSTM	0.8527	0.7470	0.7025
ECN	0.8552	0.7464	0.7256
EmoGraph2vec	0.8815↑+2.3%	0.7703↑+10.9%	0.7627↑+2.7%
**EMFSA (ours)**	**0.9016**	**0.8543**	**0.7833**

On each of the three benchmark datasets, the EMFSA model obtains the highest level of precision. The MSD_Emoji dataset yields a performance increase of 2.3%, the TD_Emoji dataset provides a performance increase of 10.9%, and the ERD_Emoji dataset demonstrates an optimal classification performance increase of 2.7%.

Among the baseline models, TextCNN and Att-BiLSTM use traditional machine learning methods such as convolutional neural networks, bidirectional long short-term memory (BiLSTM), and attention mechanisms to capture the most essential semantic information in a sentence, but their classification accuracies are poor. Even on the MSD_Emoji dataset, which has a larger emoji corpus, the highest accuracy is only 82%; on the other two datasets, which have sparse data resources, the highest accuracy achieved by these methods is only 72%. The classification accuracies of these two models are not substantially enhanced. These methods are incapable of capturing context-specific sentiment information in situations where sentiment semantics are inadequately expressed in brief texts. The EA-Bi-LSTM and ECN models, which utilize bidirectional long short-term memory networks and attention models, are better able to capture bidirectional semantic dependencies and perceive semantics. Both models yield improvements of 3 points on the MSD_Emoji dataset, with an accuracy of up to 85%, and nearly 3 points on the TD_Emoji and ERD_Emoji datasets, with an accuracy of up to 74%, compared to the conventional machine learning methods.

In 2022, the EmoGraph2vec was newly proposed; this model learns emoji representations by embedding emoji nodes within a co-occurrence graph network and enriching semantic information using the EmojiNet external knowledge base. The model derives emoji representations using both Emoji2Vec and Unicode characters; it achieves 88% accuracy on the MSD_Emoji dataset, while the accuracy increases by nearly 3 percentage points to 77% on the TD_Emoji and ERD_Emoji datasets. However, the classification effect of this method is insufficient for datasets with sparse corpora, and there is still considerable room for improving its sentiment analysis accuracy when addressing the semantic sparseness of short texts, words with multiple meanings, and diverse data modalities on social network platforms such as Twitter and Facebook.

**Fig 4:**
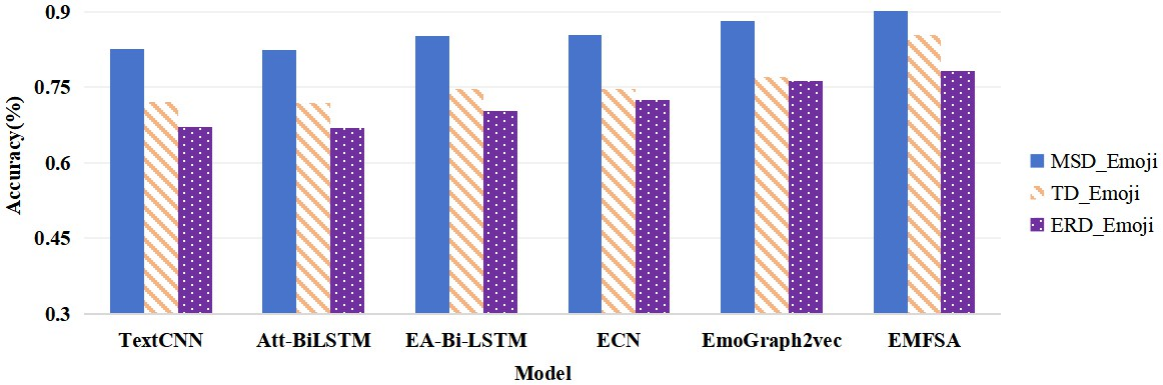
Visualization of the results of the accuracy comparison experiment conducted with the baseline models.

The EMFSA model achieves the highest classification accuracy in comparison with the baseline method when performing sentiment analysis tasks on emoji-containing text data from the three benchmark datasets. The method designed for this model can be used to effectively mine the contextual semantics of short English texts on Twitter containing emojis, better realize the complementarity of multiple features, and further improve its ability to recognize and perceive contextual sentiments by embedding emoji features. A visualization of the outcomes of the accuracy comparison experiment conducted with the baseline models is shown in Fig 4.

### Ablation experiment

To more rigorous determine the validity of the proposed model, we devise four ablation schemes, and four evaluation indices areas are utilized: the accuracy, macro-precision, macro-recall, and macro-F1 scores. The ablation experiments are described as follows:

- **W/o topic:** Topic features are not used; i.e., when multiple features are fused, the topic of each word is set to have the same degree of conformity as that of the overall topic of the text.
- **W/o fusion:** The multifeature fusion module is not used; i.e., the *h*_[*CLS*]_ output from emo_exBERT are directly input into the classifier for sentiment classification.
- **W/o senti-mlm**: In the domain-specific additional pretraining stage of emo_exBERT, the original masked language model (MLM) is used instead of our Senti_MLM.
- **W/o emoji**: Emoji information is not used, as follows: (1) Replace emo_exBERT with the BERT model and use the EmojifyData-EN dataset with the emojis removed for additional pretraining. (2) Remove all emojis from the dataset used when training the EMFSA model via the sentiment analysis task. (3) Do not use the multifeature fusion module (same as w/o fusing), as the data do not contain emojis at this point, and using this module would only introduce noise.
- **EMFSA (ours)**: This is the proposed EMFSA model.

#### Results obtained on the MSD_Emoji dataset

The results of the experiments conducted on the MSD_Emoji dataset are detailed in Table 3. Compared to the approach that does not use emoji symbol information (w/o emoji), the proposed model achieves relative performance improvements of 31.2%, 33.2%, 56.9%, and 60.3% under the four indices, accuracy, macro-precision, macro-recall, and the macro-F1 score, respectively. The first three experiments also show that the use of topic features, feature fusion, and sentiment vocabulary-prioritized masking schemes all lead to performance improvements, where the emoji features and topic features are more effective at improving the accuracy of sentiment analysis. This finding verifies the effectiveness of the sentiment analysis model proposed in this paper.

**Table 3:** Results of ablation experiments conducted on MSD_Emoji.

Model	MSD_Emoji			
	Accuracy	Macro-Precision	Macro-Recall	Macro-F1
w/o topic	0.8948 ± 0.0227	0.7240 ± 0.0109	0.6986 ± 0.0046	0.7060 ± 0.0131
w/o fusing	0.8948 ± 0.0218	0.7200 ± 0.0337	0.7062 ± 0.0096	0.7147 ± 0.0097
w/o senti-mlm	0.8948 ± 0.0194	0.7135 ± 0.0269	0.6899 ± 0.0104	0.7103 ± 0.0174
w/o emoji	0.6872 ± 0.0055	0.5528 ± 0.0142	0.4588 ± 0.0170	0.4522 ± 0.0073
**EMFSA (ours)**	**0.9016** ± 0.0283	**0.7366** ± 0.0265	**0.7199** ± 0.0172	**0.7250** ± 0.0120

#### Results obtained on the ERD_Emoji dataset

The results of the experiments conducted on the ERD_Emoji dataset are detailed in Table 4. Compared to the method that does not use emoji symbol information (w/o emoji), the model proposed in this paper achieves relative performance improvements of 46.9%, 65.3%, 40%, and 53.9% under the four metrics, accuracy, macro-precision, macro-recall, and the macro-F1 score, respectively. However, the experimental results obtained using the thematic features, feature fusion, and sentiment vocabulary-prioritized masking schemes are consistent with those of the EMFSA model; moreover, the main reason for this finding is that this dataset is small, and the model is unable to make full use of its cross-attention mechanism to extract features.

**Table 4:** Results of ablation experiments conducted on ERD_Emoji.

Model	MSD_Emoji			
	Accuracy	Macro-Precision	Macro-Recall	Macro-F1
w/o topic	0.7833 ± 0.1333	0.5530 ± 0.0581	0.5250 ± 0.0584	0.5277 ± 0.0610
w/o fusing	0.7833 ± 0.1333	0.5530 ± 0.0581	0.5250 ± 0.0584	0.5277 ± 0.0610
w/o senti-mlm	0.7833 ± 0.1333	0.5530 ± 0.0581	0.5250 ± 0.0584	0.5277 ± 0.0610
w/o emoji	0.5333 ± 0.1333	0.3345 ± 0.0359	0.3750 ± 0.0250	0.3428 ± 0.0361
**EMFSA (ours)**	**0.7833** ± 0.1333	**0.5530** ± 0.0581	**0.5250** ± 0.0584	**0.5277** ± 0.0610

#### Results obtained on the TD_Emoji dataset

The results of the experiments conducted on the TD_Emoji dataset are detailed in Table 5. Compared to those of the method that does not use emoji symbol information (w/o emoji), the four indicators exhibit relative performance improvements of 87%, 159%, 139%, and 165%. Compared with the model using thematic features and feature fusion, the relative performance improvement is greater than 2.5% on average. The macro-recall and macro-F1 metrics are slightly lower than those attained without using senti_mlm. This may be because the proportion of sentiment words included in TD_Emoji is low, so the senti_mlm mechanism is not significantly different from the normal MLM. The randomness of the actual masking process leads to better model learning of during pretraining when using the MLM.

**Table 5:** Results of ablation experiments conducted on TD_Emoji.

Model	TD_Emoji			
	Accuracy	Macro-Precision	Macro-Recall	Macro-F1
w/o topic	0.8373 ± 0.0222	0.8167 ± 0.0364	0.8030 ± 0.0213	0.8085 ± 0.0278
w/o fusing	0.8375 ± 0.0252	0.8207 ± 0.0382	0.7953 ± 0.0182	0.8040 ± 0.0247
w/o senti-mlm	0.8522 ± 0.0194	0.8289 ± 0.0311	**0.8184** ± 0.0244	**0.8229** ± 0.0260
w/o emoji	0.4568 ± 0.0417	0.3204 ± 0.0073	0.3396 ± 0.0117	0.3091 ± 0.0182
**EMFSA (ours)**	**0.8543** ± 0.0280	**0.8321** ± 0.0318	0.8131 ± 0.0226	0.8206 ± 0.0308

## Conclusion

Emojis provide important information about users’ sentiments when analyzing short informal texts such as tweets, blogs, or comments. We propose EMFSA, a sentiment analysis model with multifeature fusion based on emoticons, to address the issues of undirected sentiment expressions, “multiple meanings of words,” and the low accuracy attained by sentiment analysis methods for brief English texts on social platforms. The developed model uses a combination of multiple features, such as emoticons, themes, and texts, to sentimentally mine various modes of information and achieve intermodal information enhancement and complementation. Specifically, the model utilizes the semantic and sentiment functions of emoticons to fully mine contextual semantics, fill in visual cues in the employed text corpus, improve the ability of the model to process brief text information, and effectively express and enhance the sentiments contained in the text.

In this paper, the EMFSA model achieves optimal performance on all three public benchmark datasets, with relative accuracy improvements of 2.3%, 10.9%, and 2.7%. To further demonstrate the efficacy of emojis for the sentiment analysis task, we devise four scenarios for ablation experiments. Utilizing the MSD_Emoji dataset for illustrative purposes, the experimental results demonstrate that the EMFSA model achieves relative improvements of 31.2%, 33.2%, 56.9%, and 60.3% over an ablation model using emoji symbol information without emojis (w/o emoji) in terms of the accuracy, macro-precision, macro-recall, and macro-F1 metrics, respectively. The results of the experiments indicate that emoticons can effectively enhance the sentiments of text. In addition, the sentiment word priority masking model, the biterm topic model, and the cross-attention fusion mechanism can further improve the semantic representations of short text sentiments, thereby enhancing the accuracy achieved in short text sentiment analysis tasks.

In our future work, we will investigate a method for fusing emojis with multimodal data (video, audio, and images). Detecting sarcasm and irony in text is also a challenge in the field of natural language processing. We will investigate whether sarcasm and irony can be handled with the aid of emoticons to increase the accuracy of sentiment analysis models.
